# The adverse effect of gentamicin on cell metabolism in three cultured mammary cell lines: “Are cell culture data skewed?”

**DOI:** 10.1371/journal.pone.0214586

**Published:** 2019-04-01

**Authors:** Robert L. Elliott, Xian-Peng Jiang

**Affiliations:** The Sallie A. Burdine Breast Foundation, Baton Rouge, Louisiana, United States of America; University of South Alabama Mitchell Cancer Institute, UNITED STATES

## Abstract

Many cells are cultured in media that contains an antibiotic to prevent bacterial contamination. Mycoplasma and other bacterial contamination is a serious problem for those involved in cell culture. Antibiotics in the media helps prevent this contamination and make life easier for the investigators; as performing cell culture experiments in antibiotic free media is difficult and requires vigorous sterile technique. There are many reports of antibiotics causing mitochondrial damage. In this study, we tested the effect of gentamicin in culture media on human mammary epithelial MCF-12A and breast cancer MCF-7 and MDA-MB-231 cell lines by real time PCR, immunofluorescent microscopy, lactate assay, DNA damage assay. We found that the addition of gentamicin in media upregulated the gene expression of hypoxia inducer factor 1 alpha (HIF1a), glycolytic enzymes and glucose transporters, compared to the cells cultured in gentamicin free media. Gentamicin also increased the lactate production and inhibited mitochondrial membrane potential of the cell lines. Furthermore, the antibiotics in media induced mitochondrial reactive oxygen species causing DNA damage. We found an increase of 8-hydroxy-2’-deoxyguanosine a product of DNA oxidative damage in the media of MCF-12A, MCF-7 and MDA-MB-231 cell lines. These results showed that normal epithelial and breast cancer cells cultured in the media with gentamicin had increased HIF1a, aerobic glycolysis and DNA oxidative damage. If we use these unhealthy cells in the experiment, all data will be different, compared to cells grown in gentamicin free media. We have studied the detrimental effects of three antibiotics on mitochondrial function in the untransformed MCF-12A human mammary cell line and two human mammary cancer cell lines, MCF-7 and MB-MDA-231. The metabolic changes in all cell lines were dramatically different between those in antibiotic free media versus antibiotic containing media. There was a marked difference in gene expression of glycolytic enzymes, reactive oxygen species production and effects on membrane potential. Ironically, our first studies were done in media containing gentamicin, and repeated studies were done in gentamicin free media. The results were very different. The purpose of this report is to emphasize that metabolic cell culture data may be inaccurate because experiments were performed in cell culture media containing antibiotics. We will present evidence to support this theory.

## Introduction

The investigative discipline of cell culture has contributed tremendous research knowledge to the field of cancer and cell biology. During the past 30–40 year cell culture data led to developing many in vivo models in mice. The technique has been done in cancer cell lines to study drug sensitivity and resistance translating into clinical decisions. Many of these papers discuss in the Materials and Methods section that the cell lines were incubated with antibiotics.

It is known that bactericidal antibiotics induce mitochondrial dysfunction and oxidative damage in mammalian cells [1].This antibiotic damage to mitochondria is because they are evolutionary bacteria. Lynn Margulis stated many years ago that mitochondria were probably evolutionary bacteria that formed an endosymbiotic relationship with an eukaryotic host cell over a billion years ago [2]. Michael Gray demonstrated scientific and DNA evidence affirming a bacterial origin of mitochondria [3]. Mitochondria share similar ribosomes and protein synthesis machinery as do bacteria. Therefore, it is logical antibiotics that cause bacterial lethality could also damage mammalian mitochondria.

Some articles on good cell culture practice and guidelines for the use of cell lines in cancer research have emphasized the importance to remember that antibiotics can disrupt and arrest critical aspects of cell biology. They state where possible antibiotics should be avoided, never be routine in the cell culture laboratory and never used to replace effective aseptic techniques [4].

There are many problems associated with cell culture that are unfortunately disregarded in the medical community. This happens in biotechnology, academic research and pharmaceutical industry. Unfortunately, much scientific data has had to be modified or retracted because of these problems. This is especially true because of cross-contamination between cells especially with Mycoplasma [5, 6].

About eight years ago after years involved in cancer research, we began to study cancer metabolism and the associated mitochondrial dysfunction. We reviewed the ultrastructural morphology in 778 breast cancer specimens and noticed a marked difference in the number and ultrastructural morphology of mitochondria that correlated with the grade of the tumor. The most aggressive tumors had very few and very abnormal mitochondria [7].This led to reviewing the work of Warburg. In the 1930s, he reported that tumorigenesis was caused by mitochondrial dysfunction, and that cancer cells had defective respiration with increased glycolysis and lactate production even in the presence of oxygen. This aerobic glycolysis became known as the “Warburg Effect” [8, 9].

This led to our work of mitochondrial organelle transplantation (MOT). Our first report showed that the introduction of normal epithelial mitochondria into cancer cells inhibited proliferation and increased drug sensitivity [10]. Our second study revealed that exogenous normal mammary epithelial mitochondria suppressed glycolytic metabolism and glucose uptake of human breast cancer cells [11]. Ironically, these first studies were performed in cell culture media containing gentamicin. We are now implementing repeat experiments of these studies to be done in antibiotic free media.

There are many papers in the literature on the effects of antibiotics on metabolism of cell lines. Many papers have contradictory results even when the cell culture media was very similar and the media supposedly antibiotic free. Unfortunately, many of the experiments were performed in cell culture media containing antibiotics, while the investigators were testing the effect of another antibiotic on cell metabolism. This paradox may explain why there is a discrepancy in results, creating a need for these studies to be repeated in antibiotic free cell culture media. The inconsistency of these results will be addressed in the discussion section of this communication.

The main objective of this report is to emphasize that antibiotics can cause mitochondrial dysfunction and that cell culture studies on cell metabolism should probably be done in antibiotic free media. Previous reports on this type data should probably be repeated in antibiotic free media. Our evidence on this problem will be presented.

## Materials and methods

### Cell culture

Human immortalized, untransformed mammary epithelial cell line, MCF-12A, and human breast cancer cell lines, MCF-7 and MDA-MB-231, were obtained from American Type Culture Collection (Rockville, MD, USA). MCF-7 is estrogen receptor (ER) and progesterone receptor (PR) positive, but MDA-MB-231 is ER and PR negative. All cell lines were maintained in antibiotic-free media at 5%CO_2_ and 37°C. MCF-7 and MDA-MB-231 were grown in alpha minimum essential medium (a-MEM) supplemented with 10% fetal calf serum and 1 mM glutamine (GIBCO Invitrogen, Carlsbad, CA, USA). Immortalized, untransformed MCF-12A cells were cultured in a 1:1 mixture of Ham’s F12 medium and Dulbecco’s Modified Eagle’s Medium containing 0.1 μg/mL cholera enterotoxin, 10 μg/mL insulin, 0.5 μg/mL hydrocortisone, 20 μg/mL epidermal growth factor, and 5% horse serum (Sigma Chemical Co., St. Louis, MO, USA). Cells were detached from tissue culture flasks by digestion with 0.05% trypsin and 0.53 mM EDTA.

### Mitochondria staining for mitochondrial membrane potential detection

Six thousands of MCF-12A, MCF-7 or MDA-MB-231cells were cultured in 35mm 1.5 glass bottom dishes containing gentamicin free media overnight. The media was aspirated on the second day. The dishes were filled with media containing 0.05mg/ml gentamicin. The control was filled with the media without gentamicin. All cells were then cultured for 4 hours at 5% CO_2_ and 37°C. Mitochondria were stained with mitochondria staining kit (Sigma CS0390, St. Louis, MO, USA). The brief procedure is as follows: Mix 25μl of 200x JC-1 (5, 5’, 6, 6’-tetrachloro-1, 1’, 3, 3’-tetraethylbenzimidazolocarbocyanine iodide) Stock Solution in 4 ml of ultrapure water in a test tube. Close the test tube and mix solution by inversion or vortex test tube briefly. Incubate test tube for 2 minutes at room temperature to insure that JC-1 is completely dissolved. Open the test tube and add 1 ml of JC-1 Staining Buffer 5x. Mix by inversion. Then, mix the staining mixture with an equal volume of complete medium for cell growth. Aspirate growth medium from flask and overlay cells with the above mixture. Add 0.2–0.4 ml of the mixture per 1 cm^2^ of growth surface. Incubate cells for 20 minutes at 37°C in humidified atmosphere containing 5% CO_2_. Aspirate the mixture. Wash the cells twice with cold growth medium. Fluorescence is observed by Olympus IX83 fluorescent microscope (Tokyo, Japan). In cells which maintain electrochemical potential gradient, the dye accumulates in mitochondria, where it forms bright red fluorescent aggregates (J-aggregates). Three pictures were taken in different areas each dish. All pictures were taken with 400ms exposure. The intensity of fluorescence was measured by software Cellsens 1.16. Mean and standard deviation of the intensity is calculated.

### RT-qPCR

We used real time PCR (RT-qPCR) to measure gene expression of glycolytic enzymes and glucose transporters. The protocol is as described in detail previously [11]. MCF-12A or MCF-7 or MDA-MB-231 cells were cultured in media with 0.05mg/ml gentamicin addition at 37°C and 5% CO_2_ for 7 days. Cells cultured in gentamicin free media were as controls. Cells were removed from plates by trypsin-EDTA digestion. Total RNA was isolated by PureLink RNA Kit (Invitrogen, Carlsbad, CA). cDNA was synthesized by the High Capacity RNA-to-cDNA kit (Applied Biosystems, Grand Island, NY). In brief, 2 μg of total RNA was mixed 10μl of 2x RT buffer and 1μl of 20x Enzyme Mix and water in total 20μl of reaction volume. The reaction mixture was incubated for 60 minutes at 37°C and then 5 minutes at 95°C to stop the reaction. The cDNA was ready for use in real time PCR application or long-term storage in freezer. We examined hypoxia-inducible factor alpha (HIF1a), three glycolytic enzymes hexokinase (HK2), Phosphofructokinase-1 (PFKM) and pyruvate kinase (PKM2), glucose transporter 1 (SLC2A1 or Glut 1) and 3 (SLC2A3 or Glut 3) and lactate dehydrogenase A (LDHA) which catalyzes the reduction of pyruvate by NADH to form lactate,. All gene expression quantification was performed with TaqMan Gene Expression Assay, a proven 5’ nuclease-based real-time PCR chemistry. Primers and probes (PrimeTime Mini qPCR assay) were synthesized by Integrated DNA Technologies (IDT, Coralville, Iowa) (Table 1). Β-actin (ACTB) was used as endogenous gene control to normalized PCRs for the amount of RNA added to the reverse transcription reactions. Probes contain at the 5’ end the FAM (6-carboxy fluorescein) as a fluorescent reporter dye, and internal and at 3’ end the ZEN/Iowa Black FQ as fluorescent double quenchers. Forty μl of qPCR reaction mixture contained 20μl of TaqMan universal PCR master mix (Applied Biosystems, Grand Island, NY), 4μl of 10x PrimeTime Mini qPCR assay (IDT, Coralville, Iowa) and 16μl of cDNA (100ng). The qPCR reaction was aliquoted in triple to wells of 384-well PCR plate. The plate was sealed, briefly centrifuged, and performed reaction with 7900HT real time PCR system (Applied Biosystems, Grand Island, NY). Standard mode ran as 2 minutes at 50°C and 10 minutes at 95°C, and 40 cycles (15 seconds at 95°C and 1 minute at 60°C). Target gene expression was determined by relative quantification (RQ) which related signal of the target transcript in a treated group to that of an untreated control (medium only). We analyzed relative quantification with the RQ Manager 1.2 software (Applied Biosystems, Grand Island, NY). Gene expression was calculated as the ratio of mRNA of the cells cultured in 0.05mg/ml gentamicin media to that of the cells in gentamicin free media.

**Table 1:** List of primer and probe sequences for RT-qPCR.

Gene	Pair of primers (FWD and REV)	Probe
ACTB	GGATCAGCAAGCAGGAGTATG; AGAAAGGGTGTAACGCAACTAA	TCGTCCACCGCAAATGCTTCTAGG
HIF1a	GTCTGCAACATGGAAGGTATTG; GCAGGTCATAGGTGGTTTCT	ACTGCACAGGCCACATTCACGTAT
HK2	GCAGAAGGTTGACCAGTATCTC; CCAAGCCCTTTCTCCATCTC	CACATGCGCCTCTCTGATGAGACC
PFKM	GCATCCCATTTGTGGTCATTC; GTCACAGGTTGTGCAGATAGT	AATGTCCCTGGCTCAGACTTCAGC
PKM2	CTGTGGCTGGACTACAAGAA; CTGCTTCACCTGGAGAGAAATA	AAGTGGGCAGCAAGATCTACGTGG
LDHA	AGATTCCAGTGTGCCTGTATG; ACCTCTTTCCACTGTTCCTTATC	AGTGGAATGAATGTTGCTGGTGTCTCT
SLC2A1	CTGGGCAAGTCCTTTGAGAT; GTGACACTTCACCCACATACA	AGTACACACCGATGATGAAGCGGC
SLC2A3	AGGATGCAGGTGTTCAAGAG; GCCCTTTCCACCAGAAATAGA	CGGCGCGGGTGTGGTTAATACTAT

### Measurement of lactate

Lactate concentration in culture media was measured with Glycolysis Cell-based Assay Kit (Cayman Chemical Company, Ann Arbor, MI). The detailed method was found in the product protocol. 10,000 of MCF-12A, MCF-7 or MDA-MB-231 cells in 100μl were sub-cultured in triple on 96-well cell culture plate and cultured for 24 hours at 37°C and 5% CO_2_. Cells cultured in gentamicin free media were used as the controls. The plate was centrifuged at 1000rpm for 5 minutes. Ten μl of the supernatant from each well was transferred to corresponding wells on a new 96-well plate for lactate measurement. One hundred μl of Reaction Solution was added to each well, including the standard wells. The plate was incubated with gentle shaking on an orbital shaker for 30 minutes at room temperature and the absorbance read at 492nm with a plate reader. A standard curve was plotted by using absorbance of a series of standard. Lactate concentration of samples was calculated against the standard curve.

### Mitochondrial superoxide detection

Mitochondrial superoxide is detected by MitoSox Red mitochondrial superoxide indicator (Invitrogen, Carlsbad, CA). The MitoSox Red reagent is live cell permeant and is rapidly and selectively targeted to the mitochondria. Once in the mitochondria, MitoSox Red reagent is oxidized by superoxide and exhibits red fluorescence. 6,000 cells were cultured in 35mm 1.5 glass bottom dishes containing gentamicin free media overnight. The media were aspirated at second day. The dishes were filled with media containing 0.05mg/ml gentamicin. The control cells were cultured in media without gentamicin addition. All cells were cultured for 24 hours and stained with 5μM MitoSox Red reagent working solution for 10 minutes at 37°C. The cells were washed 3 times with Hank’s balanced salt solution and obtained fluorescence by Olympus IX83 fluorescent microscope. Three pictures were taken in different areas each dish. All pictures were taken with 2s exposure. The intensity of fluorescence was measured by software Cellsens 1.16.

### DNA oxidative damage examination

10,000 of MCF-12A, MCF-7 or MDA-MB-231 cells in 100μl were sub-cultured in triple on 96-well cell culture plate and cultured for 24 hours at 37°C and 5% CO2. Cells cultured in gentamicin free media were used as the controls. DNA oxidative damage is examined by DNA Damage Competitive ELISA Kit (Invitrogen, Carlsbad, CA). Among numerous types of oxidative DNA damage, the formation of 8-hydroxy-2’-deoxyguanosine (8-OHdG) is a ubiquitous marker of oxidative stress. 8-OHdG is quantitated by DNA Damage Competitive ELISA Kit. In brief, all components are allowed to reach room temperature before use. Add 50μl of standard samples or cell culture media to the appropriate wells of the 96-well plate pre-coated with goat anti-rabbit IgG, then 25μl of 8-OHdG and 25μl of 8-OHdG antibody. Cover the plate with plate sealer and incubate for 2 hours at room temperature with shaking. Aspirate the solution and wash wells 4 times with 300μl of 1x washing buffer. Thoroughly aspirate the washing buffer and add 100μl TMB substrate to each well. Incubate the plate for 30 minutes at room temperature without shaking. Add 50 μl of Stop Solution to each well and read the absorbance at 450nm within 10 minutes. The concentration of 8-OHdG of each sample is calculated with software Four Parameter Logistic Curve.

### Statistical analysis

Data were analyzed by t-test and presented as mean ± standard deviation (SD). Findings were considered significant at p<0.05.

## Results

### Gentamicin changes the morphology of human mammary epithelial MCF-12A

**Fig 1:**
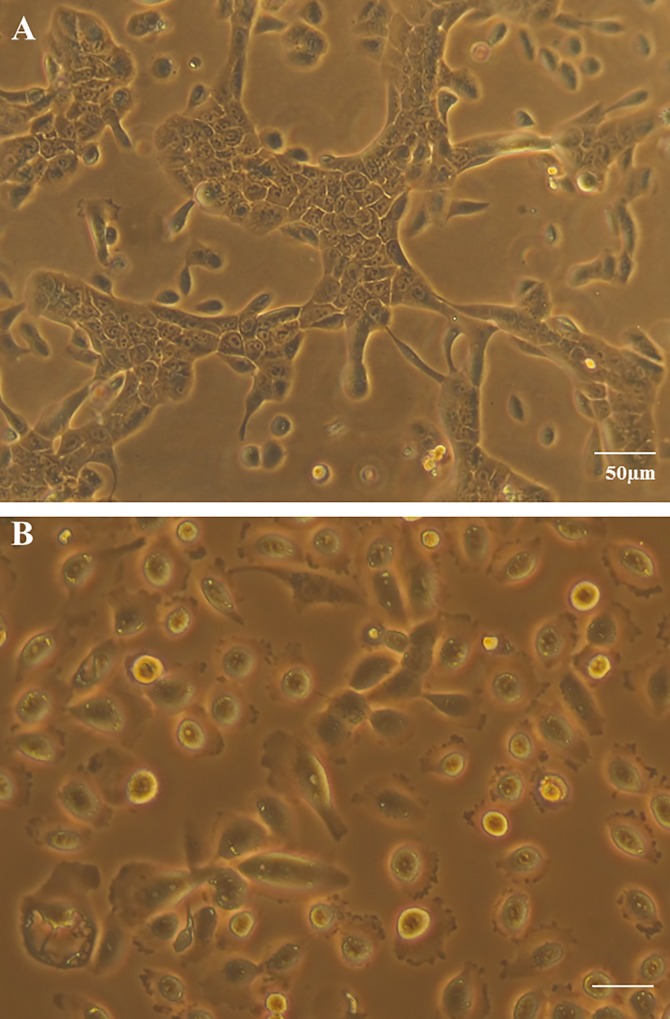
Human mammary epithelial MCF-12A cells cultured in the media without and with gentamicin. A: the medium without gentamicin; B: the medium with 0.05 mg/ml gentamicin.

MCF-12A cells previously cultured in medium with 0.05mg/ml gentamicin were switched to the gentamicin free medium. After 4–5 days of culture, the MCF-12A cells began to form a ductal pattern. However, MCF-12A cells cultured in gentamicin-added medium grew as scattered pattern (Fig 1). This is strong evidence of gentamicin toxic effect on cells in cell culture. We didn’t find significant change in the morphology of MCF-7 and MDA-MB-231 cells grown in gentamicin media.

### Addition of gentamicin inhibits mitochondrial membrane potential gradient

**Fig 2:**
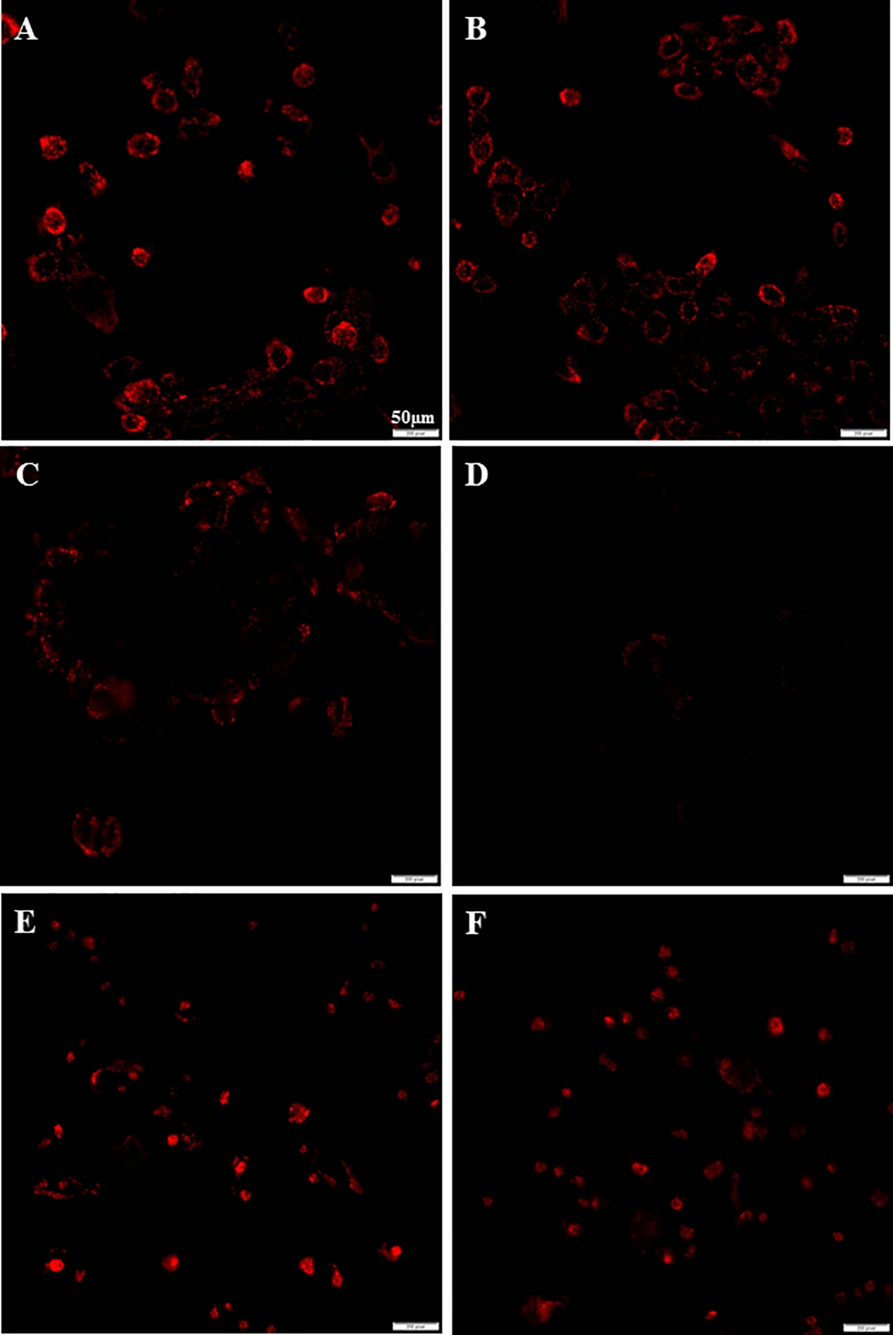
Gentamicin addition in the media inhibited mitochondrial membrane potential gradient in MCF-12A, MCF-7 and MDA-MB-231 cells. A,B: MCF-12A; C,D: MCF-7; E,F: MDA-MB-231. A, C, E: the media without gentamicin; B,D,F: the media with 0.05mg/ml of gentamicin.

Cells maintained in gentamicin free media were switched to the media with 0.05mg/ml gentamicin for 4 hours. We found that addition of 0.05mg/ml gentamicin significantly inhibited the mitochondrial membrane potential of MCF-7 cells (fluorescent intensity: 30.7±10.5), compared to the cells cultured in gentamicin free medium (55.5±10.3) (p<0.05). The 0.05mg/ml of gentamicin also suppress the mitochondrial membrane potential of MCF-12A (50.3±13.8) and MDA-MB-231 (55.6±10.3), compared to the cells in gentamicin free media (MCF-12A: 68.2±10.6; MDA-MB-231: 73.5±10.9), but the difference is not statistically significant (both p>0.05) (Fig 2).

### Gentamicin upregulates gene expression of HIF1a, glycolytic enzymes and glucose transporters

**Fig 3:**
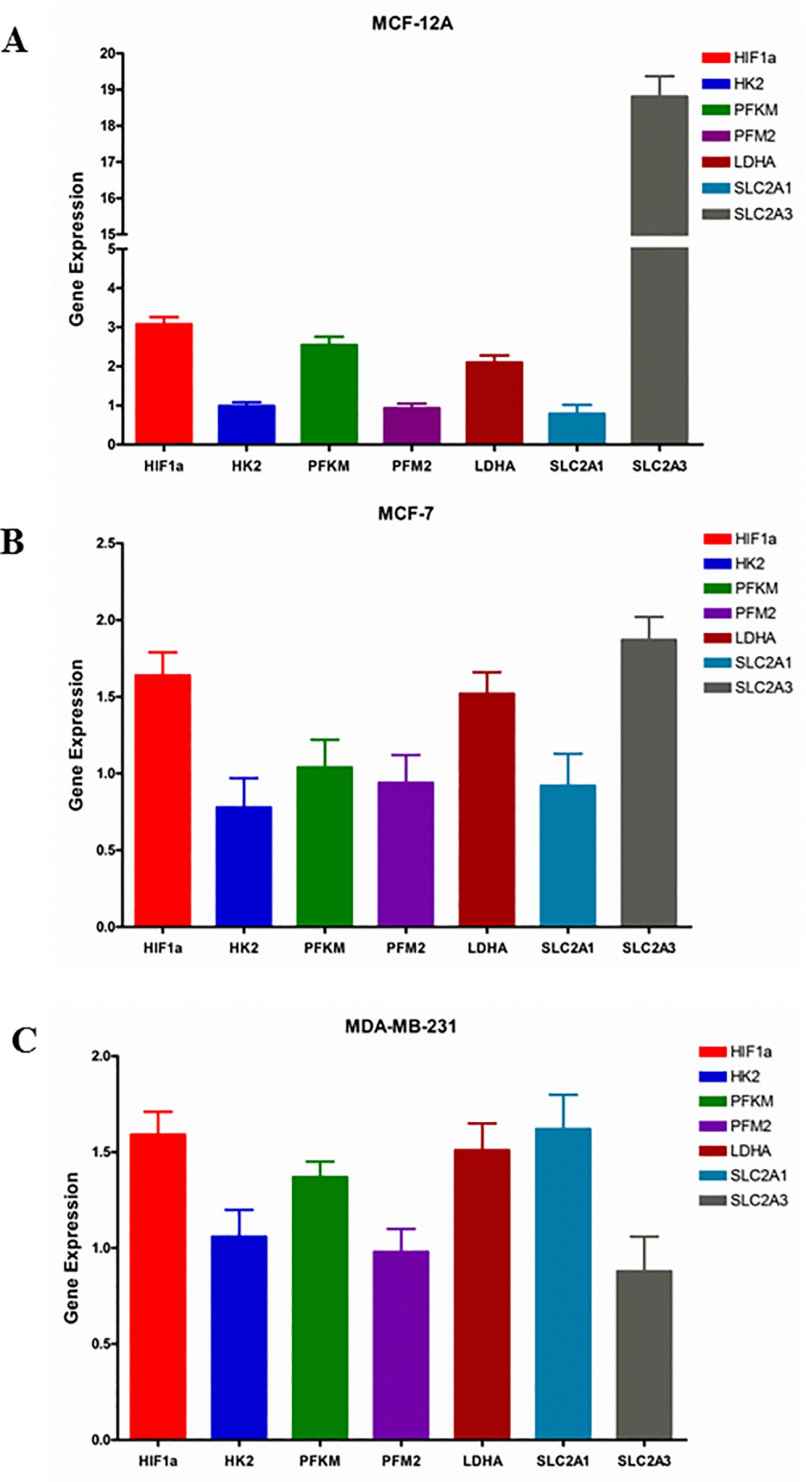
Gene expression of HIF1a, glycolytic enzymes and glucose transporters in MCF-12A, MCF-7 and MDA-MB-231 cultured in the media without and with 0.05mg/ml gentamicin addition. mRNA was measured by real time PCR. Gene expression was calculated as the ratio of mRNA of the cells cultured in the media with gentamicin to that of the cells in the media without gentamicin. A: MCF-12A; B: MCF-7; C: MDA-MB-231.

MCF-12A, MCF-7 and MDA-MB-231 maintained in gentamicin free media were sub-cultured in the media containing 0.05mg/ml gentamicin for 7 days. The cells cultured in gentamicin free media were as the controls. The mRNA expression was measured by real time PCR. Gene expression was calculated as the ratio of mRNA of the cells cultured in 0.05mg/ml gentamicin media to that of the cells in gentamicin free media. The effect of gentamicin addition in the media on gene expression is shown at Fig 3. HIF1a mRNA of MCF-12A, MCF-7 and MDA-MB-231 cell lines cultured in media with gentamicin is 3.08, 1.64 and 1.59 times that of the cell lines grown in the media without gentamicin, respectively. The addition of gentamicin in the media also increased mRNA expression of glycolytic enzyme PFKM gene of MCF-12A (2.55 times) and MDA-MB-231 (1.37 times) cells. Gene expression of LDHA, an enzyme that catalyzes the reduction of pyruvate by NADH to form lactate, was upregulated by gentamicin in three cell lines MCF-12A, MCF-7 and MDA-MB-231 (2.10, 1.52 and 1.51 times, respectively). Gentamicin increased gene expression of glucose transporters in MCF-12A (SLC2A3: 18.81 times), MCF-7 (SLC2A3: 1.87 times) and MDA-MB-231 (SLC2A1: 1.62 times) cells. All above gene upregulation of the cell lines in media containing gentamicin are statistically significant (all p<0.05), compared to that of the cell lines in gentamicin-free media. These results suggest that the addition of gentamicin in medium induces aerobic glycolysis of the cultured cells.

### Gentamicin increases lactate production

MCF-12A, MCF-7 or MDA-MB-231 cells were cultured with the media with 0.05mg/ml gentamicin for 24 hours. The concentration of lactate in the medium was significantly increased in human mammary epithelium MCF-12A (p<0.05), compared MCF-12A cells cultured in gentamicin free medium (Table 2). Gentamicin also increased the lactate concentration in the cultured media of MCF-7 and MDA-MB-231, but the difference is not statistically significant (both p>0.05) (Table 2).

**Table 2:** Lactate production of cell lines cultured in the media without or with 0.05mg/ml gentamicin addition. L-lactate concentration (μM) was measured by Cayman glycolysis cell-based assay kit.

Cell line	Amount of cells	Media without gentamicin	Media with 0.05mg/ml gentamicin	p^2^
MCF-12A	10,000	2.29±0.41(3)^1^	3.35±0.45(3)	<0.05
MCF-7	10,000	3.46±0.45(3)	4.25±0.40(3)	>0.05
MDA-MB-231	10,000	3.39±0.38(3)	4.28±0.54(3)	>0.05

^1^ Mean ± SD (N)

^2^ Unpaired t-test, less than 0.05 as significant; NS: not significant

### Gentamicin induces mitochondrial superoxide

**Fig 4:**
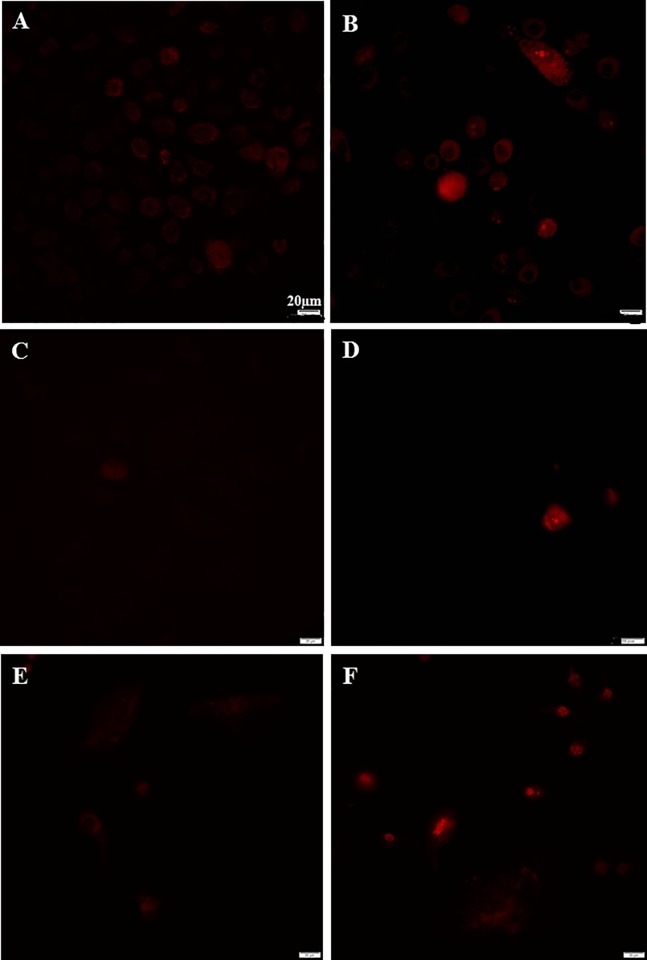
Gentamicin in culture media increases mitochondrial superoxide of MCF-12A, MCF-7 and MDA-MB-231 cells. A, C, E: the media without gentamicin; B, D, F: the media with 0.05mg/ml gentamicin. A, B: MCF-12A; C, D: MCF-7; E, F: MDA-MB-231.

The predominant reactive oxygen species (ROS) is superoxide. The mitochondrial superoxide is determined by MitoSOX red mitochondrial superoxide indicator. The intensity of fluorescence was measured by software Cellsens 1.16. The addition of 0.05mg/ml gentamicin in the media induced mitochondrial superoxide production of MCF-12A, MCF-7 and MDA-MB-231 cells after 24 hours of culture (Fig 4). The fluorescent intensity of MCF-12A (43.8±10.4), MCF-7 (36.7±9.8) and MDA-MB-231 (44.4±11.2) cells cultured in the media with gentamicin was significantly increased, compared to the MCF-12A (21.3±9.4), MCF-7 (11.7±5.7) and MDA-MB-231(18.4±8.7) cells in the media without gentamicin, respectively (p<0.05 for all cell lines).

### Gentamicin increases cell DNA oxidative damage

Cells were cultured in the media containing 0.05mg/ml gentamicin for 24 hours. The concentration of 8-OHdG in the culture medium of MCF-12A was significantly higher than that of cells in medium without gentamicin (p<0.05). The addition of gentamicin also increased the concentration of 8-OHdG in the media of MCF-7 and MDA-MB-231 cells, but the difference is not significant (p>0.05) (Table 3). These results show gentamicin in culture media increase DNA oxidative damage of cultured cells.

**Table 3:** DNA oxidative damage of cell lines cultured in the media without or with 0.05mg/ml gentamicin addition. 8-OHdG concentration (pg/mL) in the media was measured by DNA Damage Competitive assay kit.

Cell line	Amount of cells	Media without gentamicin	Media with 0.05mg/ml gentamicin	p^2^
MCF-12A	10,000	248±65(3)^1^	375±41(3)	<0.05
MCF-7	10,000	353±49(3)	445±0.46(3)	>0.05
MDA-MB-231	10,000	351±48(3)	449±0.45(3)	>0.05

^1^ Mean ± SD (N)

^2^ Unpaired t-test, less than 0.05 as significant; NS: not significant

## Discussion

The discussion of this communication will be difficult and possibly confusing. The problem is there are many reports published by good scientist that are contradictory. Many things may have caused this confusion; but we believe a major factor is the use of different cell culture media. Some studies were done in antibiotic free culture media, while others were done in culture media containing an antibiotic and then adding the antibiotic to be tested. It seems counterproductive to report findings of how an antibiotic can cause mitochondrial dysfunction when it is tested in media that already may have damaged the mitochondria. We will try to decipher the discrepancy in these important studies and relate them to our research data. It is then hoped that we may inspire investigators to establish standard reproducible laboratory techniques to obtain consistent cell culture research data. Why is this important? Well it is impossible to compare mitochondrial damage caused by a specific antibiotic if it is tested in cell culture media already containing an antibiotic to prevent contamination.

We will now discuss our results of the effect of antibiotics on mitochondrial function in human cell lines. We will compare our results done in antibiotic free culture media to those obtained in antibiotic containing cell culture media. These findings will be compared to studies of other investigators. We found in our studies that gentamicin in the culture media affected the morphology of the human mammary epithelial MCF-12A cell line. The cells were undifferentiated and scattered in the antibiotic media, but were differentiated and had a distinct ductal pattern in antibiotic free media (Fig 1). These morphological changes were not observed on the human breast cancer cell lines MCF-7 and MDA-MB-231. The addition of gentamicin to the cell culture media inhibited the mitochondrial membrane potential of both cancer cell lines MCF-7, MDA-MB-231, and also the normal MCF-12A cell line (Fig 2).

Gentamicin containing media also upregulated the gene expression of HIF-1a, glycolytic enzymes and glucose transporters in all three humans cell lines (Fig 3, S1 File). Gentamicin also increased lactate production of all three cell lines but was more pronounced and significant in the normal MCF-12A cell line. It was increased in the two cancer cell lines but was not significant (Table 2). Gentamicin induced mitochondrial superoxide in all three cell lines after 24 hours (Fig 4) and it was significant when compared to the cell lines incubated in antibiotic free media. Gentamicin also caused increased cell DNA oxidative damage. Cells cultured in media containing 0.05 mg/ml of gentamicin for 24 hours increased the concentration of 8-OHdG in the normal MCF-12A cell lines. It also increased it in the cancer cell lines but it was not significant (Table 3). These results suggest antibiotics may be more detrimental to normal cells. All of these results demonstrate that cell culture studies of cell metabolism should be done in antibiotic free media; as antibiotic media causes mitochondrial dysfunction resulting in inaccurate data. This suggests that many previous reports need to be re-evaluated.

One group reported that bactericidal antibiotics induce mitochondrial dysfunction and oxidative damage in mammalian cells. They exposed the human mammary epithelial cell line MCF-10A to three bactericidal antibiotics from three different classes: [1] ampicillin (aβ-lactam), ciprofloxacin (a fluoroquinolone), and kanamycin (an aminoglycoside). All three of them caused a dose-time dependent increase in intracellular reactive oxygen species (ROS) production. They did not see a significant increase in ROS production from a bacteriostatic antibiotic (tetracycline) [1]. However, this was not our finding with doxycycline and was not the findings of others, which will be presented later.

However, this group also found that mitochondrial dysfunction and oxidative damage could be prevented by administration of the antioxidant N-acetyl -2-cysteine. The administration of the antioxidant did not affect the efficacy on the lethality of the bacteria. This group did use an antibiotic free treatment media, however, it was unclear in the Materials and Methods section as how long the cells were out of the antibiotic culture media before being exposed to the bactericidal antibiotic [1]. It might take several weeks out of antibiotic media to reverse possible damage to cells done by the antibiotic containing culture media to prevent contamination [1].

Another group has reported on a very sophisticated study of isolated mitochondria evaluating the effects of various antibiotics on mitochondrial function. They found that at therapeutic doses ciprofloxacin did not inhibit mitochondrial function, but that other antibiotics do. They found that tetracycline and chloramphenicol inhibited protein synthesis [12]. Their results were consistent with other laboratories that demonstrated in vitro and in vivo studies on the immunosuppressive effect of tetracycline [13, 14].

Ahler, Sullivan, Cass, Braas, et al, have published a paper entitled “Doxycycline Alters Metabolism and Proliferation of Human Cell Lines.”. Their findings are quite different than those described by the other group [1]. They report that tetracycline are frequently used as mediators of inducible gene expression systems in biomedical research. They discuss many known effects on mammalian cells of tetracycline especially inhibition of the mitochondrial ribosome. This, of course can inhibit protein synthesis and mitochondrial biogenesis. In studies of human cell lines, they observed commonly used concentrations of doxycycline can change the gene expression pattern and convert metabolism to a more glycolytic phenotype. This was confirmed by reduced oxygen consumption and increased lactate production. These changes at these concentrations were also sufficient to slow proliferation. They emphasize that researchers using doxycycline in an inducible system need to develop appropriate controls to account for the effect of the drug on cellular metabolism [15]. Another great well done paper described many detrimental effects of doxycycline on mitochondrial function. They observed impaired protein synthesis, shift from oxidative metabolism to glycolysis. The main effect was inhibiting translation at the ribosome inhibiting protein synthesis [16]. Ironically, the Ahler paper describes in the cell culture section of Materials and Methods that their multiple human cell lines were in cell culture media that contained penicillin and streptomycin. Those are bactericidal antibiotics that are known to cause mitochondrial dysfunction and changes in cellular metabolism.

Doxycycline is a bacteriostatic antibiotic that supposedly causes no mitochondrial oxidative damage according to Ref.[1], while others have found it to slightly increase (ROS) and promote glycolysis. However, how can we be sure the effects of doxycycline on cell lines in the Ahler paper are accurate when the experiment was done on cells incubated with penicillin and streptomycin? These cells may have already been affected with mitochondrial oxidative damage before being treated with doxycycline. Therefore, it is possible the effect of doxycycline was potentiated in cells that were already sick because of the time they were incubated in antibiotic containing media. These results are a perfect example of why cell culture studies of the effects of antibiotics on cell metabolism should be done in antibiotic free media. We believe these cells also should have been in antibiotic free media for at least two weeks before conducting the experiments.

In 2004, a paper entitled “Doxycycline induces Apoptosis in PANC-1 Pancreatic Cancer Cells” was published by another group. They evaluated doxycycline in both the in vitro and in vivo setting, and cells were never incubated in antibiotic containing cell culture media before introduction of doxycycline to test its effect on these cells. There have been many reports that tetracycline like doxycycline produced cytotoxic effect against mammalian tumor cells, though the mechanism of these effects on cell proliferation remains to be determined. These investigators set out to determine some of the mechanisms of this anti-proliferative effect. They studied the antitumor effect of doxycycline on the human pancreatic cell line PANC-1. To their credit in their experiments the cell line was not incubated with antibiotic cell culture media. They determined that cytotoxic effects of doxycycline were associated by G15 cell cycle arrest and DNA fragmentation in these cells. The drug also activated transcription of p 53, p 21 and the Fas/Fasl- cascade- related genes, and reduced expression of Bcl-xL and Mc1.They also showed in a mouse xenograft model that doxycycline treatment suppressed tumor growth by 80%. Those in vitro results can be relied upon as the cell line was not damaged by media containing an antibiotic [17]. These findings indicate more studies should be done to determine if doxycycline is synergistic with anticancer drugs or as a drug sensitizer and adjunct to chemotherapy.

Other investigators studied the effect of eight different antibiotics on inhibition of mammalian protein synthesis. The studies were done on isolated mitochondria from rat and rabbit heart, rat liver and rabbit bone marrow. Tetracycline was the most inhibitory on mitochondrial protein synthesis [18].

We have demonstrated in this presentation that antibiotics cause mitochondrial dysfunction, and have emphasized this in previous publications [19, 20]. Different groups of antibiotics cause mitochondrial damage by different mechanisms, but it is absolute that antibiotics cause mitochondrial damage. This damage may promote tumorigenesis and neurodegeneration. However, the main purpose of this communication is to ensure we can depend on in vitro data evaluating this problem. We believe that much of the published data on the effect of antibiotics on mammalian cell metabolism may be skewed; as cells were incubated in antibiotic media prior to being treated with the antibiotic under investigation.

Our results were very different in the two media suggesting many of the previous reports should be repeated. We are convinced antibiotics cause mitochondrial dysfunction and oxidative damage. However, they could possibly be used be used to treat cancer in humans as an adjunct to chemotherapy, radiation and cancer immunotherapy.

Previous data obtained in antibiotic media deserves scrutiny and probably should be repeated in antibiotic free media. Much more work needs to be done studying the effects of antibiotics on mitochondrial function, cell metabolism, and the intestinal microbiome. The latter is very important for the efficacy of cancer immunotherapy especially with check point inhibitors. We need to explore mechanisms of how antibiotics might be used as an adjunct to cancer therapy.

The possibility that antibiotics be used in cancer therapy is supported by a recent paper by Tan, Yon, Sog, and Li, et al. These investigators have shown that doxycycline enhances the sensitivity of glioblastoma to chemotherapy by induction of mitochondrial dysfunction and oxidative damage [21]. Others have shown that doxycycline decreased the tumor burden of human breast cancer in a bone metastasis model [22], while others demonstrated that antibiotics can effectively eradicate cancer stem cells across multiple tumor types by targeting mitochondria [23]. We also must evaluate in detail the paradox of how over usage of antibiotics might contribute to tumorigenesis, mitochondrial diseases and neurodegeneration. We also emphasize that all cell culture experiments on cell metabolism should be done in antibiotic free cell culture media.

## Conclusion

In summary, we believe that the main message and purpose of this communication has been accomplished and presented fairly. Based on our study of the effect of gentamicin in three mammary cell lines, we are convinced that antibiotics do cause mitochondrial dysfunction, and this is true for bactericidal and bacteriostatic antibiotics regardless of paradoxical reports in the literature. We have emphasized the importance of cell culture studies being done in antibiotic free culture media; especially when studying cellular metabolism. We have stressed that many reports may have inaccurate data, as the study was done in antibiotic containing cell culture media. When studying mitochondrial function, we must remember that mitochondria are evolutionary bacteria; and antibiotics have damaging effects on them and bacteria. Hopefully, this presentation will lead to better cell culture practice, lesson antibiotic abuse, and promote better antibiotic treatment protocols. If this occurs, we will have more than met the goal of this presentation.

## Supporting information

S1 File Gentamicin addition in culture media inhibited mitochondrial membrane potential, upregulated gene expression of glycolytic enzymes and induced DNA oxidative damage of cell lines MCF-12A, MCF-7 and MDA-MB-231. (PDF)
