# Supplementary material for: The adverse effect of gentamicin on cell metabolism in three cultured mammary cell lines: “Are cell culture data skewed?”
Source: PLoS One. 2019 Apr 1;14(4):e0214586. doi: 10.1371/journal.pone.0214586 (PMC6443165; doi:10.1371/journal.pone.0214586)

Table 1. List of primer and probe sequences for RT-qPCR.

Gene	Pair of primers (FWD and REV)	Probe
ACTB	GGATCAGCAAGCAGGAGTATG; AGAAAGGGTGTAACGCAACTAA	TCGTCCACCGCAAATGCTTCTAGG
HIF1a	GTCTGCAACATGGAAGGTATTG; GCAGGTCATAGGTGGTTTCT	ACTGCACAGGCCACATTCACGTAT
HK2	GCAGAAGGTTGACCAGTATCTC; CCAAGCCCTTTCTCCATCTC	CACATGCGCCTCTCTGATGAGACC
PFKM	GCATCCCATTTGTGGTCATTC; GTCACAGGTTGTGCAGATAGT	AATGTCCCTGGCTCAGACTTCAGC
PKM2	CTGTGGCTGGACTACAAGAA; CTGCTTCACCTGGAGAGAAATA	AAGTGGGCAGCAAGATCTACGTGG
LDHA	AGATTCCAGTGTGCCTGTATG; ACCTCTTTCCACTGTTCCTTATC	AGTGGAATGAATGTTGCTGGTGTCTCT
SLC2A1	CTGGGCAAGTCCTTTGAGAT; GTGACACTTCACCCACATACA	AGTACACACCGATGATGAAGCGGC
SLC2A3	AGGATGCAGGTGTTCAAGAG; GCCCTTTCCACCAGAAATAGA	CGGCGCGGGTGTGGTTAATACTAT

Table 2. Lactate production of cell lines cultured in the media without or with 0.05mg/ml gentamicin addition. L-lactate concentration (μM) was measured by Cayman glycolysis cell-based assay kit.

Cell line	Amount of cells	Media without gentamicin	Media with 0.05mg/ml gentamicin	p[2]
MCF-12A	10,000	2.29±0.41(3)[1]	3.35±0.45(3)	<0.05
MCF-7	10,000	3.46±0.45(3)	4.25±0.40(3)	>0.05
MDA-MB-231	10,000	3.39±0.38(3)	4.28±0.54(3)	>0.05

[1] Mean ± SD (N)

[2] Unpaired t-test, less than 0.05 as significant; NS: not significant

Table 3. DNA oxidative damage of cell lines cultured in the media without or with 0.05mg/ml gentamicin addition. 8-HOdG concentration (μM) in the media was measured by DNA Damage Competitive assay kit.

Cell line	Amount of cells	Media without gentamicin	Media with 0.05mg/ml gentamicin	p[2]
MCF-12A	10,000	248±65(3)[1]	375±41(3)	<0.05
MCF-7	10,000	353±49(3)	445±0.46(3)	>0.05
MDA-MB-231	10,000	351±48(3)	449±0.45(3)	>0.05

[1] Mean ± SD (N)

[2] Unpaired t-test, less than 0.05 as significant; NS: not significant

Fig. 1. Human mammary epithelial MCF-12A cells cultured in the media without and with gentamicin. A: the medium without gentamicin; B: the medium with 0.05 mg/ml gentamicin.

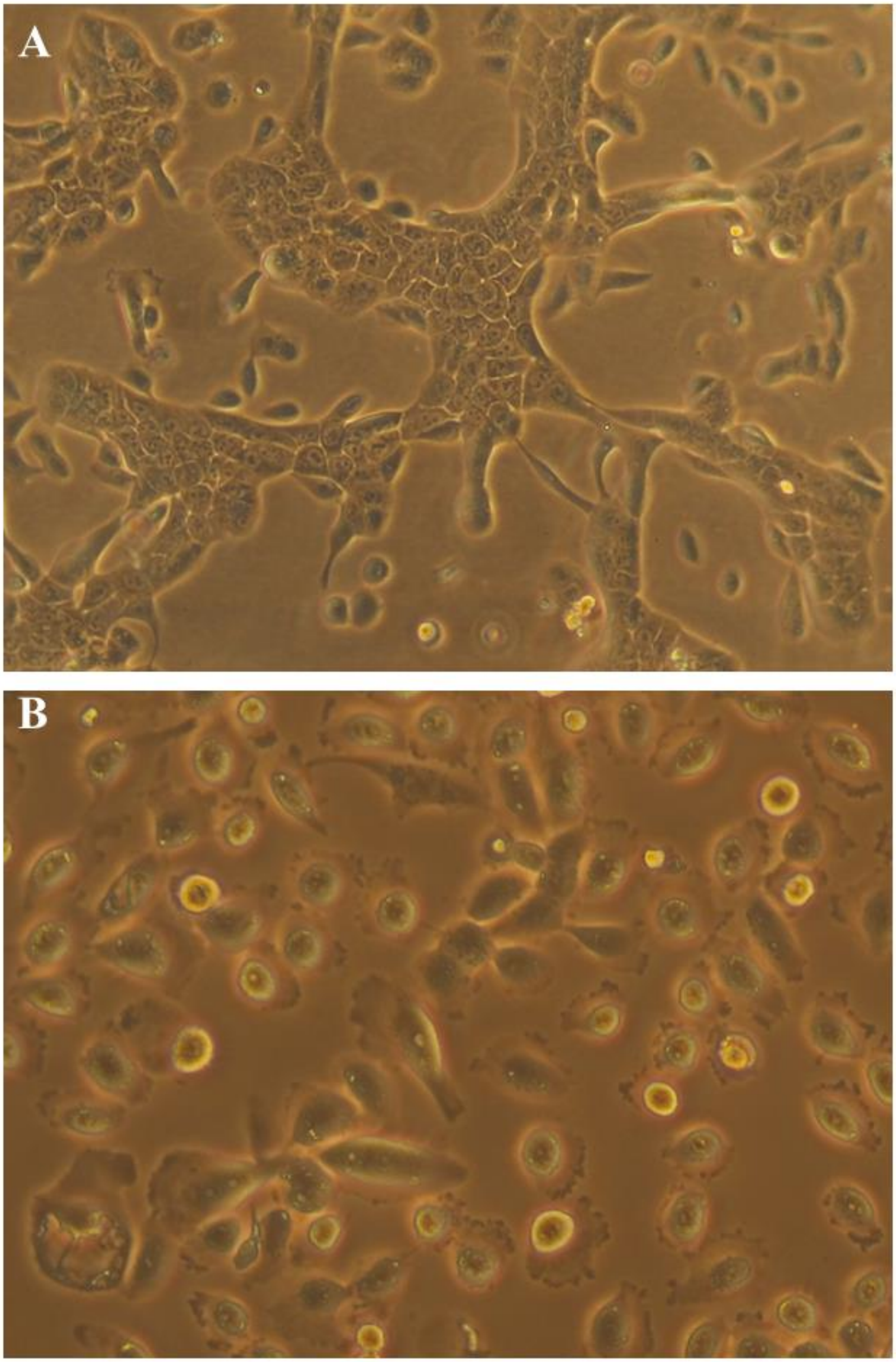

Fig. 2. Gentamicin addition in the media inhibited mitochondrial membrane potential gradient in MCF-12A, MCF-7 and MDA-MB-231 cells. A,B: MCF-12A; C,D: MCF-7; E,F: MDA-MB-231. A, C, E: the media without gentamicin; B,D,F: the media with 0.05mg/ml of gentamicin. (The details seen in the Materials and Methods)

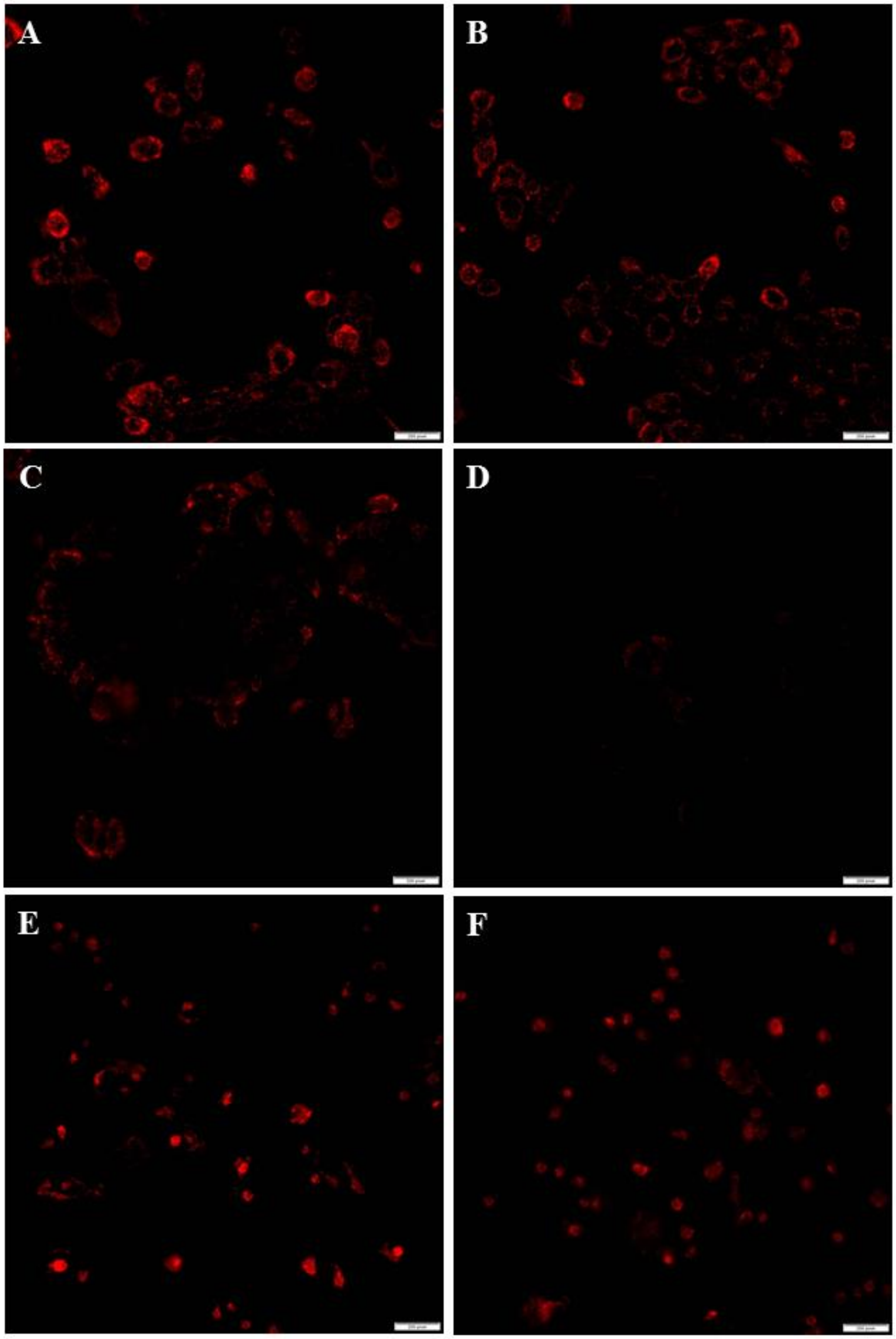

Fig. 3. Gene expression of HIF1a, glycolytic enzymes and glucose transporters in MCF-12A, MCF-7 and MDA-MB-231 cultured in the media without and with 0.05mg/ml gentamicin addition. mRNA was measured by real time PCR. Gene expression was calculated as the ratio of mRNA of the cells cultured in the media with gentamicin to that of the cells in the media without gentamicin. A: MCF-12A; B: MCF-7; C: MDA-MB-231.

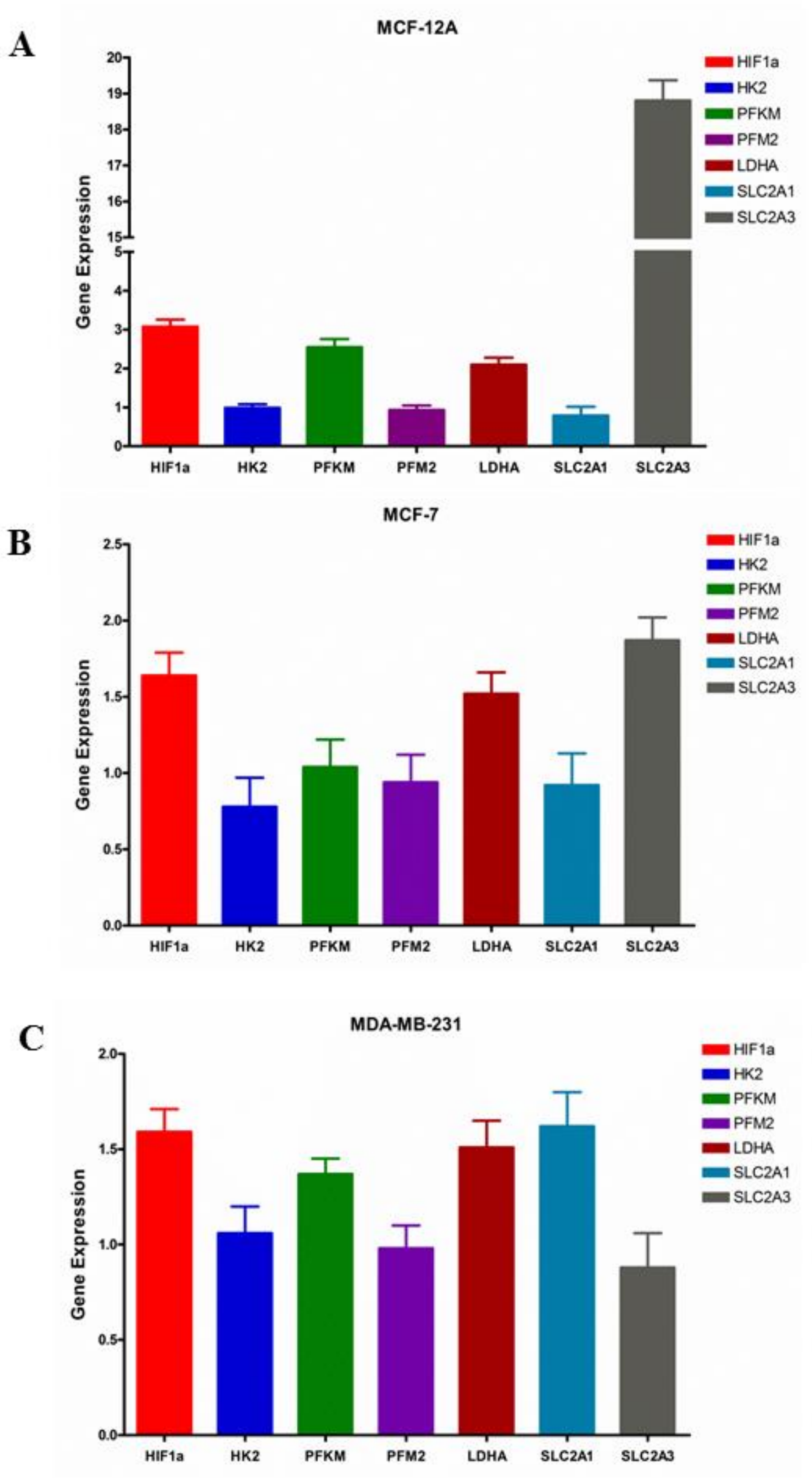

Gene expression data for Fig.3:

Cell lines	HIF1a*	HK2	PFKM	PFM2	LDHA	SLC2A1	SLC2A3
MCF-12A	3.08±0.18(3)	0.99±0.09(3)	2.55±0.21(3)	0.93±0.12(3)	2.10±0.18(3)	0.79±0.23(3)	18.81±0.56(3)
MCF-7	1.64±0.15(3)	0.78±0.19(3)	1.04±0.18(3)	0.94±0.18(3)	1.52±0.14(3)	0.92±0.21(3)	1.87±0.15(3)
MDAMB231	1.59±0.12(3)	1.06±0.14(3)	1.37±0.08(3)	0.98±0.12(3)	1.51±0.14(3)	1.62±0.18(3)	0.88±0.18(3)

*Mean±SD (N)

Fig. 4. Gentamicin in culture media increases mitochondrial superoxide of MCF-12A, MCF-7 and MDA-MB-231 cells. A, C, E: the media without gentamicin; B, D, F: the media with 0.05mg/ml gentamicin. A, B: MCF-12A; C, D: MCF-7; E, F: MDA-MB-231.

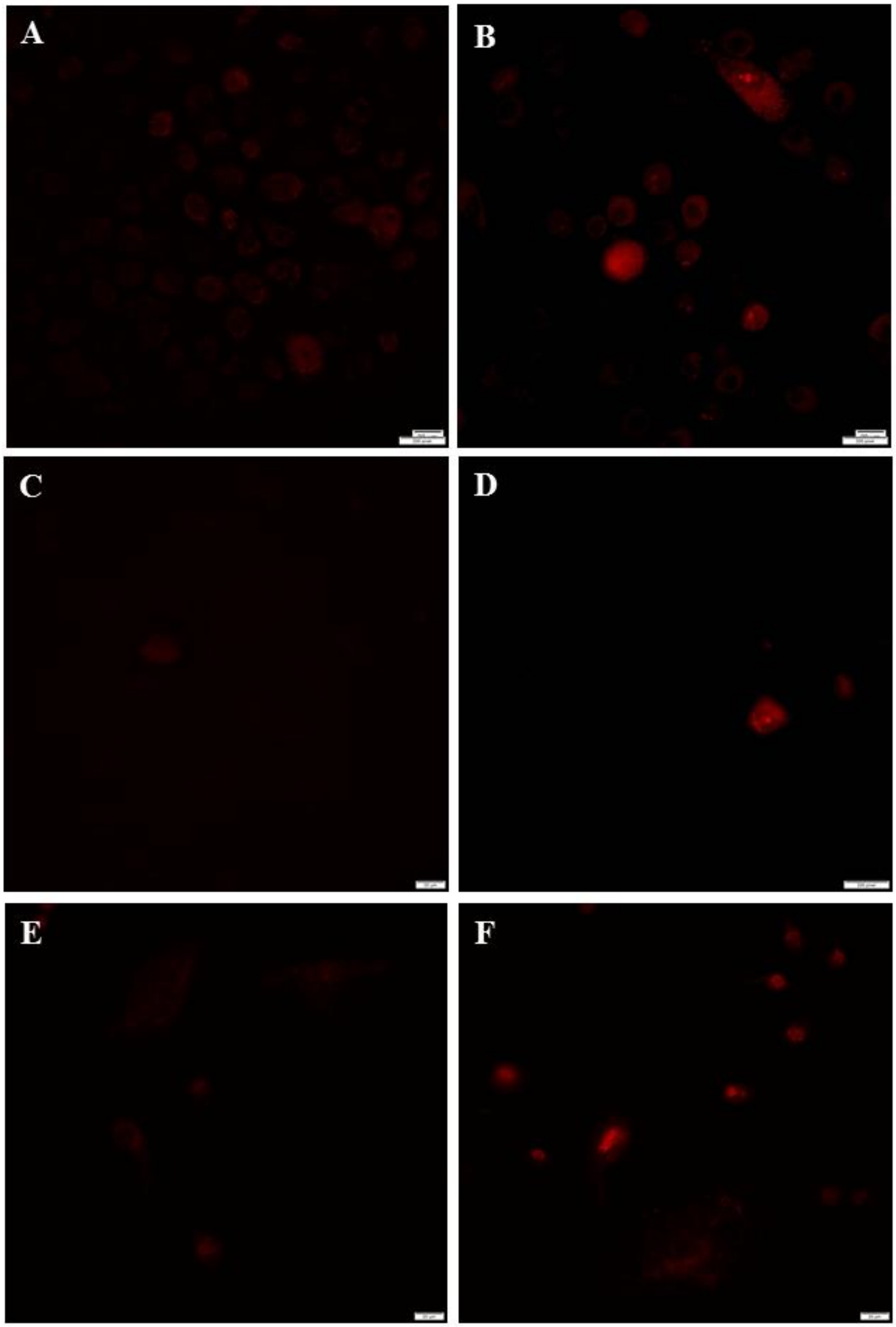

Supplement: S1 File — (PDF) [file pone.0214586.s001.pdf]
